# Obesity-Related Atrial Fibrillation: Cardiac Manifestation of a Systemic Disease

**DOI:** 10.3390/jcdd10080323

**Published:** 2023-07-30

**Authors:** Worawan B. Limpitikul, Saumya Das

**Affiliations:** 1 Cardiovascular Research Center, Massachusetts General Hospital, Harvard Medical School, Boston, MA 02114, USA; wlimpitikul@mgh.harvard.edu; 2 Demoulas Family Foundation Center for Cardiac Arrhythmias, Massachusetts General Hospital, Boston, MA 02114, USA

**Keywords:** atrial fibrillation, obesity, metabolic syndrome, adiposity, atrial myopathy, atrial remodeling

## Abstract

Atrial fibrillation (AF) is the most common arrhythmia worldwide and is associated with increased morbidity and mortality. The mechanisms underlying AF are complex and multifactorial. Although it is well known that obesity is a strong risk factor for AF, the mechanisms underlying obesity-related AF are not completely understood. Current evidence proposes that in addition to overall hemodynamic changes due to increased body weight, excess adiposity raises systemic inflammation and oxidative stress, which lead to adverse atrial remodeling. This remodeling includes atrial fibrosis, atrial dilation, decreased electrical conduction between atrial myocytes, and altered ionic currents, making atrial tissue more vulnerable to both the initiation and maintenance of AF. However, much remains to be learned about the mechanistic links between obesity and AF. This knowledge will power the development of novel diagnostic tools and treatment options that will help combat the rise of the global AF burden among the obesity epidemic.

## 1. Introduction

Atrial fibrillation (AF) is the most common arrhythmia in adults. It is associated with increased morbidity and mortality due to an increased risk of stroke, heart failure, death [1], increased hospitalization [2], and decreased quality of life [3]. AF constitutes a significant public health and economic burden. AF-related healthcare costs account for about 1% of the National Health Service budget in the United Kingdom [4] and between 6 and 26 billion dollars of annual healthcare spending in the United States [5,6]. The etiology of AF is multifactorial, encompassing genetic, environmental, and lifestyle-related risk factors. Most notably, obesity is known to be strongly associated with increased risk of AF, while strategies to induce weight loss may be associated with decreased risk of future AF. However, the mechanistic underpinning of obesity-related AF remains incompletely understood and is evolving. This review aims to provide a comprehensive synthesis of the current understanding of the mechanisms underlying obesity-related AF and discuss potential future directions of AF research.

## 2. Epidemiology of Obesity and AF

The prevalence of AF worldwide is around 33.5 million patients, and it affects 2.5–3.5% of all individuals across the globe [7]. The AF burden is high across races and sexes. For example, the lifetime risk of AF in adults is 36% in Caucasian men, 30% in Caucasian women, 21% in African American men, and 22% in African American women [8]. Although there is slightly more limited data on the Asian population, a nationwide cohort in Taiwan showed a 16% and 14% lifetime risk of AF in men and women, respectively [9]. The incidence of AF is also predicted to double, from 1.2 million cases in 2010 to 2.6 million cases in 2030, while the prevalence is projected to increase from 5.2 million in 2010 to 12.1 million cases in 2030 [10]. Importantly, as one-third of the total AF population is asymptomatic [11], these numbers are likely to be underestimated.

The key question is what drives the increase in the AF burden. Although improved detection rates in the era of consumer-driven usage of smartphones and wearables may partly explain this increase [12], changes in intrinsic characteristics of the population likely remain the major driver of increased AF prevalence and incidence. For example, as age is one of the strongest risk factors for AF [13,14], the aging of the overall population significantly contributes to the increased prevalence of AF. Among modifiable risk factors (hypertension, diabetes mellitus, obstructive sleep apnea, myocardial infarction, and heart failure), obesity is strongly associated with AF.

Obesity is defined by the World Health Organization as increased adiposity or excessive accumulation of fat that may impair health. Clinically, multiple metrics are used to quantify and define obesity. A commonly used metric is body mass index (BMI). For adults, a BMI of 25.0–29.9 kg/m^2^ indicates overweight, while a BMI over 30 kg/m^2^ indicates obesity. Three classes of obesity, by BMI, include classes I, II, and III for BMI of 30.0–34.9, 35.0–39.9, and 40 kg/m^2^ or higher, respectively. Additionally, obesity can be measured by waist circumference (≥102 cm in men and ≥88 cm in women in the Caucasian populations, and ≥90 cm in men and ≥80 cm in women in South Asian, Chinese, and Japanese populations [15]), waist-to-hip ratio, skin fold thickness, and percentage of body fat, etc. [16,17,18]. There is much debate about which metrics are better for defining obesity [19]. However, regardless of the metrics used to define obesity, there is a consistent trend of increased mortality with an increased degree of adiposity [20]. For example, a meta-analysis of 57 prospective studies with 900,000 adults showed that there was a 30% increase in overall mortality per 5 BMI units [21]. Unfortunately, due to changes in diet and lifestyle in modern society, the worldwide prevalence of overweight and obesity has increased significantly over the past decades. It is estimated that by 2030, one in three adults will be overweight, and one in five adults will be obese [22]. Additionally, a rapid rise in the prevalence of childhood obesity, which has increased eightfold over the past four decades in children aged 5–19 years old [23], will surely worsen the prevalence of obesity in future adult populations.

There are multiple lines of evidence linking obesity and AF. Numerous epidemiological studies have shown that obesity is associated with new-onset AF even after adjustment for other comorbidities associated with obesity [24,25,26]. A meta-analysis of 51 studies with over 600,000 individuals by Wong et al. showed a 29% greater risk of overall incident AF and a 10% greater risk of postoperative AF for each 5-unit increase in BMI [19,27]. Additionally, obesity is also associated with the progression of AF (change in AF type to more advanced types) [28,29]. For instance, a prospective cohort study of 3248 patients with a median follow-up period of 5.1 years demonstrated that class I obesity (BMI 30–34.9 kg/m^2^) is associated with a 54% increased risk of progression from paroxysmal to permanent AF [29]. Moreover, obesity increases the rate of recurrence after AF ablation [27,30] and cardioversion [31]. Specifically, there was a 13% increase in AF recurrence after an AF ablation and a 10% increase in recurrence after cardioversion per 5 units increase of BMI, as shown by a meta-analysis by Wong et al. [27] and an observational study of 2518 patients (a sub-study of the Atrial Fibrillation Follow-Up Investigation of Rhythm Management (AFFIRM) trial) [31], respectively.

Additionally, several Mendelian randomization studies have shown an association between obesity and AF. For instance, a cohort study of over 51,646 adults of European ancestry showed an association between BMI and incident AF [32]. Another Mendelian randomization study by Larsson et al. also demonstrated an association between BMI and AF in over 300,000 individuals of White British descent in the UK Biobank [33]. Moreover, there is an association between childhood obesity and AF in adults. A Mendelian randomization study of over 63,000 children showed that a higher BMI in childhood is associated with an increased risk of AF in adults [34]. Interestingly, an analysis by Zhou et al., which pooled data from 6 genome-wide association studies, demonstrated that the risk of AF in adulthood increases with earlier onset of obesity. They found that a 1 standard deviation increase in adult obesity, childhood BMI, and birth weight was associated with a 13%, 18%, and 26% increased risk of AF in adulthood, respectively [35]. These interesting findings may thus point to a causal relationship between obesity and AF.

Therefore, with the increase in the obesity burden and its association with AF, it is important to understand the mechanisms underlying obesity-associated AF. These understandings are a critical step in devising strategies for the treatment and prevention of AF.

## 3. Conceptual Models of AF

To understand the pathophysiology underlying obesity-related AF, we must first understand how AF is initiated and maintained.

There are multiple theories regarding the mechanisms of AF, dating back to the early 1900s. Classical models of AF include rapidly firing atrial ectopic foci, single re-entry circuits, and multiple functional re-entrant circuits [36]. Over the past several decades, the multiple re-entrant circuit theory has been the dominant conceptual model of AF. In the re-entry circuit model, there must be (1) triggers to initiate and (2) vulnerable substrates to sustain the circuits [37]. AF triggers are mainly located in the pulmonary veins and originate from sleeves of the atrial muscle that extend from the left atrium (LA) several centimeters into the pulmonary veins [38]. Ectopic activities that initiate re-entry circuits can be caused either by triggered activity or automaticity. There are two types of triggered activities: early afterdepolarizations (EADs) and delayed afterdepolarizations (DADs). EADs are spontaneous depolarization of the cell membrane during phases 2 and 3 of action potentials and are caused by abnormal openings of calcium channels or sodium channels. DADs are spontaneous depolarization during phase 4 of action potentials and are caused by spontaneous releases of calcium from an internal calcium store into cytosol, which leads to activation of the sodium-calcium exchanger. On the other hand, automaticity is caused by diastolic depolarization from a net inward current during phase 4 of the action potentials, most commonly from the funny current (HCN channels). Unlike cells of the sinoatrial node, which spontaneously initiate action potentials by diastolic depolarization, mature atrial and ventricular myocytes do not express HCN channels and thus lack this capability. In normal atrial tissue, the most common mechanism of ectopic activities that initiates AF is DADs. In animal studies, EADs became important only in the background of existing long-QT syndrome from abnormal sodium channel function [39] while automaticity is more likely to contribute to initiation of AF in an end-stage heart failure where current from HCN channels is upregulated as the atrial tissue reverts to a fetal phenotype [39].

To understand how vulnerable atrial substrate allows the perpetuation of AF, we will discuss two proposed models of the re-entrant circuits. One is the “leading circle” hypothesis, where a functional re-entry establishes itself in a circuit the size of the wavelength (WL). Here, WL is a product of conduction velocity (CV), how fast the action potential propagates from cells to cells, and refractory period (RP), the period immediately following stimulation during which cardiomyocytes cannot respond to further stimulation. In this hypothesis, the number of re-entrant circuits is a function of the atrial size and the WL of the re-entry circuits. For example, atrial dilation, a common finding in atria facing chronic volume and/or pressure overload, can support more re-entrant circuits compared to normal-sized atria. Additionally, a shorter WL, either from decreased CV or decreased RP, allows more re-entrant circuits to be sustained in the atria of the same size. Ultimately, having more circuits makes simultaneous termination of all circuits more difficult and thus increases the chance of AF to continue [36]. The second model is the “spiral wave” hypothesis, where maintenance of re-entry spirals depends on tissue excitability, propagation strength, RP, and the angle of curvature of the wavefront (the greater wavefront curvature, the slower CV). For instance, lower excitability and slower propagation (e.g., the use of a sodium channel blocker) limit the wavefront curvature, which mandates larger spirals [40,41].

In AF, there are both electrical and structural remodeling of atria that increase the propensity for the initiation and maintenance of AF. We will next discuss the dysregulation of adipocytes in the obese state and how this maladaptation leads to atrial electro-anatomical remodeling.

## 4. Adipose Tissue Biology

### 4.1. The Basics of Adipose Tissue

There are three major types of adipose tissue in mammals, each with unique physical and biochemical properties. These are brown, white, and beige adipose tissue. Brown adipose tissue (BAT) accounts for approximately 4% of total body fat. It resides mainly in the supraclavicular, para-aortic, para-vertebral, and supra-renal regions. The main function of BAT is thermogenesis. Therefore, brown adipocytes are small, multilocular, contain a large number of mitochondria, and express uncoupling protein-1, which allows heat generation by uncoupling the mitochondrial electron transport chain, thereby blocking ATP production and dissipating energy in the form of heat [42].

White adipose tissue (WAT), on the other hand, is the most abundant type of adipose tissue in the human body. The main function of WAT is storing energy in the form of triglycerides and secreting hormones that regulate hunger/satiety, metabolism, and inflammatory response. White adipocytes are bigger than brown adipocytes. They contain large fat droplets and few mitochondria. WAT can be subdivided into subcutaneous (SAT) and visceral (VAT) adipose tissue. While SAT occupies the space just beneath the layer of the skin, VAT resides deeper and wraps around internal organs, including the heart and blood vessels [42]. Compared to SAT, VAT is more vascularized and innervated [43]. It is also more metabolically active and plays a major role in energy balance and plasma glucose homeostasis. Adipocytes in VAT express more receptors to glucocorticoids, a key regulatory hormone for serum glucose levels via modifying metabolic adaptations during stress, such as fasting and starvation. VAT is also more sensitive to catecholamine-induced lipolysis due to the increased function of β_3_-adrenergic receptors [44]. Additionally, VAT contains more immune cells, including macrophages, which are key regulators of a chronic low-grade systemic inflammatory state in obesity. A special type of VAT includes epicardial fat, which lies directly over the surface of the myocardium with no intervening fascial plane and shares the same microcirculation as the epicardium. Additionally, epicardial adipose tissue also contains ganglionated plexi, an intrinsic cardiac nervous system that directly innervates the myocardium. Its unique position allows epicardial fat to exert direct influence on underlying atrial tissue.

The third type of adipose tissue is beige or brown-in-white adipose tissue. Beige adipocytes are more similar to brown adipocytes in that they contain multilocular lipid droplets and numerous mitochondria. However, they reside inside WAT depots. In humans, it is postulated that beige adipocytes are derived from the “browning” of white adipocytes in response to stimulation of β_3_-adrenergic receptors, for example, during chronic cold exposure or exercise [45].

### 4.2. Remodeling of Adipose Tissue during Obesity

In a state of positive energy balance, such as obesity, adipose tissue undergoes several remodeling processes to increase its lipid storage capacity. Adipocytes in WAT undergo hypertrophy (increase in cell size) of existing mature adipocytes and hyperplasia (increase in cell number) of resident adipocyte precursors. Adipocyte hypertrophy that exceeds angiogenesis capacity creates areas of local hypoxia, leading to cell necrosis and subsequently recruitment of macrophages and other immune cells [46]. These immune cells adopt proinflammatory phenotypes in an obese state, creating a chronic low-grade systemic inflammatory state leading to a spectrum of metabolic abnormalities, including insulin resistance, type 2 diabetes mellitus, and fatty liver disease [47,48]. In addition to cellular changes, the extracellular matrix surrounding these cells also has excess deposition of collagen, elastin, and fibronectin. These changes lead to increased fibrosis and thus rigidity of the extracellular matrix, which physically limits the expansion of adipocyte mass. As obesity progresses, the maximal lipid storage capacity is exceeded, and lipid storage shifts to ectopic sites, such as the viscera, heart, and vasculature [49].

## 5. Mechanisms Underlying Obesity-Related AF

The pathophysiology of obesity-related AF is multifactorial and remains incompletely understood. One approach to conceptualizing this complex process is to use the framework of AF mechanisms (Section 3) and consider changes that increase the propensity of AF initiation (triggers) and/or create a more vulnerable substrate for AF maintenance.

### 5.1. Hemodynamic Changes

To keep up with the metabolic demand from excess body weight, obese individuals have increased circulating blood volume and thus increased cardiac output via increased stroke volume [15,50]. Furthermore, the left ventricle (LV) in obese individuals with commonly associated hypertension also faces an additional elevation of an afterload. Chronic increases in wall tension from both increased pressure and chamber dilation lead to LV hypertrophy and LV diastolic dysfunction. Chronically stiff LV and elevated LV filling pressure ultimately cause increased LA filling pressure and LA dilation. This chronic LA stretch leads to a release of atrial natriuretic peptide, angiotensin II, endothelin 1, and transforming growth factor-β, inflammatory cytokines that ultimately lead to atrial myocyte hypertrophy and atrial fibrosis [51]. Overall atrial remodeling has a synergistic effect in promoting AF. That is, atrial dilation can support more re-entry circuits while atrial fibrosis causes a reduction of CV and thus WL, which also permits more re-entrant circuits to be sustained in the atrium.

In addition to directly altering hemodynamics, obesity also increases the risk of developing AF via obstructive sleep apnea (OSA), a highly prevalent sleep disorder among obese individuals [52]. OSA is strongly associated with AF [53]. Repetitive forced inspiration against a closed glottis results in a sudden drop in the intrathoracic pressure, which is transmitted to the LA. Repetitive LA stretch may result in remodeling of LA itself and pulmonary vein ostia [54], which are the most common sites of AF triggers [38]. Additionally, intermittent hypoxemia and hypercapnia can result in sympathetic activation, which increases the frequency of atrial arrhythmia triggering the initiation of AF [55].

### 5.2. Roles of Adipokines

Adipokines are paracrines secreted by adipocytes. In addition to controlling homeostasis of serum glucose levels and lipid metabolism, these adipokines also modulate the degree of systemic inflammation and could affect atrial remodeling and thus the risk of AF. There are numerous studies investigating the role of various adipokines in obesity-related AF, although some with conflicting results. Here, we will briefly discuss several key adipokines with the most evidence in relation to AF. Further details of clinical studies describing the association between various adipokines and AF can be found in a meta-analysis by Agbaedeng et al. [56].

Adiponectin is the most abundant adipokine in the body. It has anti-inflammatory and anti-fibrotic effects and is considered “cardioprotective” to vascular endothelial and cardiomyocytes [57]. A series of 100 patients undergoing cardiac surgeries demonstrated that increased epicardial fat volume, measured by cardiac computed tomography (CT), was associated with decreased expression of adiponectin in epicardial adipose tissue [58], hinting at the role of adiponectin in atrial remodeling and perhaps AF. However, there are mixed findings in terms of the relationship between serum adiponectin and AF. For example, a case-control study of 84 individuals with AF and 84 individuals in sinus rhythm showed that individuals with AF had significantly higher levels of serum adiponectin than those in sinus rhythm [59]. A sub-analysis of a prospective randomized AVOCADO (Aspirin Vs./Or Clopidogrel in Aspirin-resistant Diabetics inflammation Outcomes) trial with 273 type 2 diabetic participants demonstrated that participants with AF at baseline (12%) had higher levels of serum adiponectin compared to those without AF at baseline [60]. On the other hand, a case-control study of 80 patients hospitalized with AF and 165 controls showed no correlation between the absolute level of serum adiponectin and AF, but showed a negative correlation between the serum level adiponectin normalized with fat mass or visceral adipose tissue index and AF [61]. Lastly, an analysis of a community-based prospective cohort, the Cardiovascular Health Study (CHS), of 3,190 participants with a median follow-up of 11.4 years and 28% incident AF showed that incident AF was associated with an elevated level of serum adiponectin measured at the baseline visit [62]. Conflicting observations of the association between serum adiponectin levels and AF among various studies, even after adjusting for confounding factors, could indicate nuances of incompletely understood roles of this adipokine in AF. For example, the discrepancy of adiponectin level in serum versus epicardial adipose tissue may hint at a potentially larger influence of epicardial fat depots, which share the same microcirculation as underlying cardiomyocytes, compared to other VAT in AF.

Similarly, current evidence for the role of leptin in AF remains conflicting. In a diet-induced obese mouse model, leptin mediates atrial fibrosis and AF [63] and a higher level of serum leptin is associated with obesity [64]. However, there are conflicting reports from observational studies on the association between serum leptin levels and AF. For instance, a case-control study of 80 patients with AF showed an increased level of serum leptin compared to the control [61] while a sub-analysis of a prospective cohort of 273 patients with type 2 diabetes mellitus showed no association between serum leptin level and AF [60].

Apelin, a known cardioprotective adipokine, has been shown in an animal study to reduce angiotensin II-induced atrial fibrosis and atrial fibrillation [65]. In humans, a lower serum level of apelin is associated with an increased risk of developing postoperative AF, as observed in a retrospective case-control study of 508 patients who underwent off-pump coronary artery bypass graft (CABG) surgeries [66]. Moreover, a lower serum level of apelin is shown to be associated with AF recurrence after an intervention. For example, in a series of 93 patients with persistent AF who underwent cardioversion, individuals with lower levels of apelin at baseline had a higher rate of AF recurrence at the 6-month follow-up [67]. Another series of 61 patients with AF who underwent pulmonary vein isolation showed that patients with lower serum levels of apelin at baseline had a higher rate of AF recurrence at 6 months post-procedure [68]. Interestingly, comparing 200 obese women to 200 lean women, Zaki et al. found that obese women had a higher level of serum apelin compared to their lean counterparts [69]. Additionally, a cross-sectional study of 740 patients who underwent weight loss from bariatric surgeries showed a lower level of messenger RNA expression of apelin in omental and subcutaneous adipose tissue compared to their baseline level [70]. This finding may raise the possibility of a compensatory increase of leptin in patients with AF to counteract cardiac remodeling from obesity and requires further studies. Given the potential therapeutic targets of adipokines in obesity-induced AF, further studies are warranted.

### 5.3. Oxidative Stress

In a chronic state of excess energy balance, where the lipid storage capacity of fat depots is exceeded, there is an elevated level of circulating free fatty acids and triglycerides. In this state, cardiomyocytes have an increased uptake of free fatty acids, leading to increased fatty acid β-oxidation [71]. Additionally, elevated plasma level of free fatty acids also increases expression of mitochondrial uncoupling protein-3 [72], which leads to further increase in mitochondrial fatty acid oxidation and thus increased production of reactive oxygen species (ROS) [73]. This overall increase in oxidative stress leads to both structural and electrical remodeling of atrial tissue. For example, diet-induced obesity is associated with activation of endoplasmic reticulum oxidative stress, which leads to atrial fibrosis and increased AF inducibility in a mouse model [74]. High oxidative stress also leads to alteration of intracellular calcium homeostasis and alteration of CV, which will be further discussed in the following section.

### 5.4. Roles of Epicardial Fat in Obesity-Related AF

As discussed previously, the unique anatomical location of the epicardial fat depot with a shared circulation with the underlying epicardium allows epicardial adipose tissue to exert direct mechanical and chemical influences on underlying atrial tissue. Obese individuals have an increased volume of epicardial fat, as evidenced by an autopsy series dating back to 1933, which showed increased excess of epicardial fat in 136 obese individuals [75]. Moreover, an observational study of obese (n = 99) and lean (n = 96) patients with heart failure with preserved ejection fraction showed that epicardial fat thickness measured by transthoracic echocardiogram was 20% thicker in obese patients compared to lean individuals [76].

Multiple studies have shown that increased epicardial fat thickness and/or volume measured by various imaging modalities is associated with an increased incidence of AF. For example, two observational studies using a subset of the Framingham Heart Study cohort showed that increased epicardial fat volume measured by CT was associated with 40% higher odds of prevalent AF (n = 3217) [77] and abnormal atrial conduction (n = 1946) [78]. Additionally, a case-control study of 53 adults with AF and 52 adults without AF showed that the odds of AF increase by 42% for each milliliter increase of epicardial fat volume measured by cardiac magnetic resonance imaging (MRI), even after adjusting for LA volume [79]. A meta-analysis of 63 observational studies, including MRI, CT, and echocardiography, showed that a one standard deviation increase in epicardial fat volume was associated with 2.6-fold higher odds of AF [80]. Additionally, a series of 115 patients undergoing AF ablation showed that atrial remodeling, including lower voltage, slower CV, and greater fractionation of electrograms, was more pronounced in the regions of the atria adjacent to epicardial adipose tissue [81].

The mechanistic links between epicardial fat and AF are likely multifactorial. In addition to the potential effects of adipokines similar to those secreted from other VAT depots, epicardial adipose tissue poses several other mechanisms due to its unique location relative to atrial myocytes. For example, the proximity of epicardial fat allows direct mechanical compression of the atria [82]. Moreover, excess adiposity can lead to the direct infiltration of epicardial adipose tissue into atrial tissue. This is observed in both obesity-induced animal models [83] and in humans. In a series of 92 patients undergoing CABG surgeries, analysis of atrial tissue samples demonstrated significant fibro-fatty infiltration in tissue from patients with AF compared to those without AF [83]. Additionally, an autopsy series of 30 individuals showed increased fatty infiltration of atrial tissue in patients with a history of AF compared to controls [84]. Interestingly, in a series of 90 adults in sinus rhythm, the amount of intra-atrial fat measured by cardiac MRI monotonically increased with the growing risk of developing AF as measured by the ARIC risk score [85]. Ultimately, this fatty infiltration leads to atrial fibrosis and gap-junction remodeling with subsequent reduction of atrial CV and increase in conduction heterogeneity, which promote re-entry circuits [86].

### 5.5. Atrial Tissue Electro-Anatomical Remodeling in Obesity

**Figure 1:**
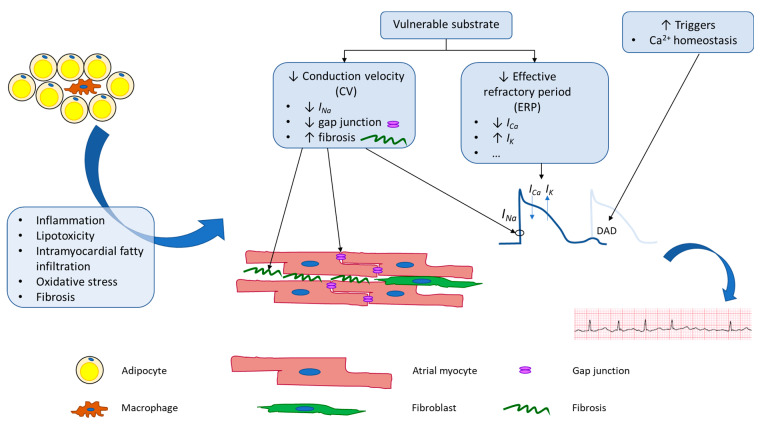
Schematic of potential mechanisms underlying adiposity-related atrial fibrillation. Obesity leads to both electrical and anatomical remodeling of atrial tissue. These changes lead to increased AF triggers and create vulnerable substrate that is more prone to perpetuation of AF.

Physiologic changes brought on by obesity alter both the electrical and structural properties of atrial tissue at a molecular level, which ultimately create a substrate primed for the initiation and maintenance of AF (Figure 1 and Table 1).

In terms of AF initiation or triggers, obesity is linked to increased oxidative stress in cardiomyocytes. In this state, ROS activates calcium/calmodulin-dependent protein kinase II (CaMKII), which leads to hyperphosphorylation of ryanodine receptors (RyR) on the sarcoplasmic reticulum (SR), a major intracellular calcium store [87,88]. In addition to increased phosphorylation, RyR is oxidized in a high oxidative stress environment [89]. Both changes lead to increased calcium leak from the SR, creating more DADs, a major source of triggers for AF initiation.

In terms of vulnerable substrates, studies conducted in various animal models demonstrated that diet-induced obese animals have increased LA fibrosis [90,91,92,93], increased atrial CV heterogeneity [94,95,96], decreased atrial RP [91,92], and thus, overall increased AF inducibility [90,91,92,93]. At the molecular level, atrial fibrosis is linked to multiple signaling pathways. For example, it is linked to increased expression of cadherin-11 [90], an adhesion molecule secreted by adipose tissue fibroblasts with major roles in the regulation of atrial tissue elasticity via altering collagen and elastin synthesis [97] and regulation of adipose tissue inflammation in obesity [98]. In a series of 43 patients undergoing CABG surgeries, expression of cadherin-11 in LA tissue excised intraoperatively was the highest in obese patients with AF, followed by lean patients with AF, and patients in normal sinus rhythm [90]. Moreover, atrial fibrosis is also associated with decreased expression of myeloid differentiation protein 1 (MD1), a key regulator in atrial modeling in response to inflammation [99]. Additionally, atrial fibrosis and inflammation are also associated with serum glucocorticoid kinase 1 (SGK1), a kinase molecule downstream of both insulin and mineralocorticoid signaling pathways [100]. Diet-induced obese mice with dominant-negative SGK1 demonstrated a reduction of AF fibrosis and proinflammatory signaling, while obese mice with constitutively active SGK1 had increased expression of proinflammatory and fibrotic molecules, compared to obese wild-type mice. Overall, atrial fibrosis leads to a reduction in CV and increased CV heterogeneity, both of which increase AF inducibility and maintenance.

A similar disturbance in electrical conduction has also been observed in humans. For example, in 212 patients without AF at baseline who underwent cardiac surgeries, intraoperative epicardial electrical mapping of the right and left atria showed more conduction delay and a higher incidence of postoperative AF in obese compared to lean patients [101]. Interestingly, conduction delay was the most severe in pulmonary veins and the Bachmann bundle, an inter-atrial connection that ensures bi-atrial synchronous contraction. In addition to atrial fibrosis, CV reduction observed in diet-induced obese animal models is associated with decreased voltage-gated sodium current (*I_Na_* or Na_V_1.5), which is responsible for phase 0 (upstroke phase) of action potentials [102] and decreased expression of connexin 40 and 43, key proteins in gap junctions [90].

Lastly, electrical remodeling of atrial tissue in obese individuals also includes alteration of ion channel expression and/or membrane current density, which ultimately alters WL and thus AF inducibility. The atrial effective refractory period (ERP) is a term used in clinical electrophysiological studies and is defined as the longest interval between two pacing stimuli that fails to capture the atrium. In a series of 63 patients with AF undergoing catheter ablation, obese patients were found to have shorter ERP compared to lean patients [103]. At the cellular level, ERP spans phases 1, 2, and 3 of cardiac action potentials. The duration of phases 1, 2, and 3 is determined by the balance between the efflux of various types of potassium currents and the influx of calcium currents. A reduction in ERP was observed in multiple diet-induced animal models and was associated with an increase in ultra-rapid delayed rectifier potassium current (*I_Kur_* or K_V_1.5) [91,102,104,105], increase in rapid and slow delayed rectifier potassium current (*I_Kr_* or K_V_11.1 or hERG and *I_Ks_* or K_V_7.1 or KCNQ1) [104], increase in transient outward potassium current (*I_to_* or K_V_4.2/4.3) [105], and decrease in L-type calcium current (*I_Ca,L_* or Ca_V_1.2) [102,104]. Overall, a reduction in CV and RP leads to a reduction in WL and thus an increased probability of AF maintenance.

## 6. Reversibility of Obesity-Induced AF and Remodeling

Multiple studies have demonstrated an association between weight loss and AF burden and progression. An observational study of 1,415 patients with AF who achieved weight loss after participation in a physician-led lifestyle modification program showed that long-term sustained weight loss was associated with maintenance of sinus rhythm [106], reduction of AF-associated symptoms [107], and delay in progression of AF from paroxysmal to persistent [15,108]. Another observational study showed that 239 morbidly obese patients who underwent bariatric surgery with resultant weight loss prior to AF catheter ablation had a lower rate of AF recurrence than those who did not undergo bariatric surgery [109]. A meta-analysis of patients with weight loss, either by lifestyle modification or bariatric surgery, showed a dose-response reduction in the frequency of AF recurrence after AF ablation [110]. Mechanistically, this may be explained by atrial reverse remodeling. In an obese sheep model, weight reduction was associated with decreased atrial fibrosis, increased atrial ERP, improved CV, decreased CV heterogeneity, and decreased AF inducibility [111]. As obesity plays a critical role in incident and recurrent AF, the reversibility of this process has significant public health implications as the rate of obesity continues to grow worldwide.

**Table 1:** Summary of studies with plausible mechanisms of obesity-related atrial remodeling.

Studies	Models	Results	Potential Signaling Pathways Involved
Mahajan et al., 2015 [95]	Sheep ± calorie-dense diet	- Obese (vs. lean): ↑ LA volume, ↑ LA pressure, ↓ CV, ↑ CV heterogeneity, ↔ ERP, ↔ ERP heterogeneity, ↑ AF burden, ↑ epicardial fat infiltrate in posterior LA and ↓ voltage in this region, ↑ atrial fibrosis and TGF-β	TGF-β (profibrotic)
Mahajan et al., 2021 [111]	Sheep ± calorie-dense diet ± weight loss (by decreasing diet intake)	- Sustained obesity (vs. lean): ↑ LA pressure, ↑ inflammation, ↑ atrial TGF-β, ↑ endothelin-B receptor expression, ↑ atrial fibrosis, ↑ epicardial fat infiltration, ↓ ERP, ↓ Cx43 and ↓ CV, ↑ CV heterogeneity, ↑ duration of induced AF - 30% weight loss (vs. obese) had ↓ LA pressure, ↓ inflammation, ↓ endothelin-B receptor expression, ↓ atrial fibrosis, ↑ ERP, ↑ Cx43 and ↑ CV, ↓ CV heterogeneity, ↓ duration of induced AF	TGF-β (profibrotic) Endothelin-1 (profibrotic)
Fang et al., 2021 [90]	Human LA tissues from patients in sinus rhythm vs. obesity + AF vs. lean + AF	- ↑ Cad-11 in LA tissue: obesity + AF > lean + AF > sinus rhythm	Cad-11 (profibrotic, proinflammatory) via MAPK-κB and NF-κB
	Mice ± high-fat diet; WT vs. Cad-11^−/−^	- Obese WT (vs. lean WT): ↑ LA diameter, ↑ LA fibrosis, ↑ Cad-11 expression in LA, ↑ p-wave duration on ECG, ↓ Cx40 and Cx43 expression, ↑ lateralization of Cx, ↑ AF inducibility, and duration - Obese Cad-11^−/−^ (vs. obese WT): ↓ LA diameter, ↓ LA fibrosis, ↓ expression of proinflammatory cytokines, ↑ Cx40 and Cx43 expression, ↓ AF inducibility and duration	
Scott et al., 2021 [91]	Human RAA tissue from obese vs. lean patients	- Obese (vs. lean): ↑ NLRP3 inflammasome activation in RAA tissue	NLRP3 inflammasome (proinflammatory) increases IL-1β and activates CaMKII which phosphorylates SR
	Sheep ± calorie-dense diet	- Obese (vs. lean): ↑ NLRP3 inflammasome activation, ↑ AF inducibility and duration, ↑ ERP	
	Mice ± high-fat diet; WT vs. NLRP3^−/−^	- Obese WT (vs. lean WT): ↑ AF inducibility and AF duration, ↓ EP, ↓ APD, ↑ *I_Kur_*, ↑ calcium leak frequency from SR, ↑ atrial fibrosis - Obese NLRP3^−/−^ (vs. obese WT): ↓ AF inducibility, ↓ *I_Kur_*, ↓ calcium leak frequency from SR	
Otsuka et al., 2021 [92]	Dogs ± high-fat diet ± rapid RA pacing	- Obese (vs. lean): ↑ AF inducibility, ↑ epicardial fat size, ↑ myocardial fat infiltrate, ↑ atrial tissue interstitial fibrosis	Not discussed
Okumura et al., 2015 [93]	Pigs ± high-fat diet	- Obese (vs. lean): ↑ LA pressure, ↓ ERP of PVs, ↑ duration of induced AF	Not discussed
Abed et al., 2013 [94]	Sheep ± calorie-dense diet	- Obese (vs. lean): ↑ LA volume, ↑ LA fibrosis, ↑ LA inflammatory infiltrates, ↑ myocardial fat infiltrate, ↓ CV, ↔ ERP, ↑ endothelin-A and -B receptors, ↑ endothelin-1	Endothelin-1 (profibrotic)
Shuai et al., 2019 [99]	WT vs. MD1^−/−^ mice + high-fat diet	- Obese MD1^−/−^ (vs. obese WT): ↑ LA fibrosis, ↑ IL-1β, IL-6, and TNF-α (proinflammatory), ↓ IL-10 (anti-inflammatory), ↑ AF inducibility	MD1 via TLR4/NF-κB pathway (proinflammatory)
Bapat et al., 2022 [100]	Mice ± high-fat diet; WT vs. SGK1^−/−^ vs. SGK1^CA^	- Obese WT (vs. lean WT): ↑ SGK1 expression, ↑ PACs on continuous telemetry, ↑ AF inducibility, ↔ CV, ↔ APD, - Obese SGK1^−/−^ (vs. obese WT): ↓ AF inducibility, ↓ APD, ↔ *I_Na_* current density, rightwards/depolarizing shift of *I_Na_* inactivation kinetics, ↓ atrial fibrosis, ↓ atrial proinflammatory signaling (↓ NLRP3, ↓ IL-1β), ↓ collagen-I expression - Obese SGK1^CA^ (vs. obese WT): ↑ PACs on continuous telemetry, ↑ AF inducibility, ↑ APD, ↑ CV, ↑ NLRP3 expression, ↑ collagen-I expression	SGK1 (part of insulin signaling and mineralocorticoid pathways)
McCauley et al., 2020 [102]	Mice ± high-fat diet ± mitochondrial antioxidant	- Obese (vs. lean): ↑ AF inducibility, ↓ APD, ↓ *I_Na_* expression and current density, ↓ *I_Ca,L_* expression and current density, ↑ *I_Kur_* expression and current density, ↑ atrial fibrosis, ↑ F2-IsoPs (↑ oxidative stress), ↑ NOX2 expression, ↑ PKC-α/δ expression - Mitochondrial antioxidant: ↓ AF burden, ↑ *I_Na_*, ↓ *I_Kur_*, ↑ *I_Ca,L_*, ↑ APD, reverse atrial fibrosis	Mitochondrial oxidative stress
Martinez-Mateu et al., 2019 [104]	Guinea pigs ± high-fat diet	- Obese (vs. lean): ↑ *I_Kr_*, + *I_Ks_*, ↓ *I_Ca,L_* current density, ↔ *I_K1_*	Not discussed
Zhang et al., 2016 [105]	Mice ± high-fat diet	- Obese (vs. lean): shorter PR-interval on ECG, ↑ SAN recovery time, ↓ ERP, ↑ *I_Kur_* expression, ↑ *I_to_* expression	Not discussed

Abbreviations: ↑, increased; ↓, decreased; ↔, unchanged; ^−/−^, homozygous knockout; ^CA^, constitutive activation; LA, left atrium; CV, conduction velocity; (E)RP, (effective) refractory period; AF, atrial fibrillation; TGF-β, transforming growth factor-β; Cx, connexin; WT, wild-type; Cad-11, cadherin-11; ECG, electrocardiogram; MAPK-κB, mitogen activated protein kinase-κB; NF-κB, nuclear factor-κB; RAA, right atrial appendage; NLRP3, NACHT, LRR, and PYD domains-containing Protein 3; APD, action potential duration; *I_Kur_*, ultra-rapid delayed rectifier potassium current; IL, interleukin; SR, sarcoplasmic reticulum; CaMKII, calcium/calmodulin-dependent protein kinase II; PVs, pulmonary veins; MD1, Myeloid differentiation protein 1; TNF-α, tumor necrosis factor-α; TLR4, Toll-like receptor-4; SGK1, serum glucocorticoid kinase 1; PACs, premature atrial complexes; *I_Na_*, voltage-gated sodium channel; *I_Ca,L_*, L-type voltage-gated calcium channel; F2-IsoPs, F2-isoprostanes; NOX2, NADPH oxidase 2; PKC, protein kinase C; *I_Kr_*, rapid delayed rectifier potassium current; *I_Ks_*, slow delayed rectifier potassium current; *I_K1_*, inwardly rectifying potassium current; SAN, sinoatrial node; *I_to_*, transient outward potassium current.

## 7. Perspective on Future Areas of Research

While the AF burden continues to rise, there are currently limited tools available for the diagnostics and treatment of AF. Although the gold standard of AF diagnosis is an electrocardiogram (ECG), paroxysmal AF may be missed in a routine 10-s 12-lead ECG or even a continuous heart monitor with a maximal duration of approximately two weeks. In patients with high suspicion for AF, for example, those with cryptogenic stroke, one may choose to obtain a longer-term recording (up to 5 years) using an implantable loop recorder, which involves a semi-invasive procedure and thus is without risk. Alternatively, biomarkers obtained non-invasively from serum or other secretions, such as saliva or urine, can prove to be an invaluable tool to predict incident AF and/or prognosticate AF progression. There has been a tremendous effort to identify various biomarkers specific to AF, including serum microRNAs, other non-coding RNAs [112,113], proteins/hormones [114,115], and metabolites [116,117]. However, the accuracy of these biomarkers is not yet sufficient for day-to-day clinical usage.

In terms of therapeutics, two current options are antiarrhythmics and AF ablation. Though non-invasive, utilization of antiarrhythmics is not without risk, as most of these medications are also pro-arrhythmic. Additionally, a widely utilized potent antiarrhythmic amiodarone has both short- and long-term pulmonary, thyroid, and liver toxicity. AF ablation involves intentionally creating scars to electrically isolate pulmonary veins, the most common site of AF triggers, from the left atrial tissue. Although more effective than antiarrhythmic medications in maintaining normal sinus rhythm, AF ablation is invasive, with complication rates up to 2.5% even in medical centers with experience in AF ablation [118]. Most importantly, although pulmonary vein isolation is very effective in AF treatment, it does not treat the root cause of AF, which is atrial myopathy, and AF can recur when atria become sicker over time.

Therefore, more effort should be focused on discovering novel diagnostic and therapeutic strategies for AF. A potentially appealing target is extracellular vesicles (EVs). EVs are lipid bilayer-bound particles released from cells and contain numerous cargos, including DNA, RNA, proteins, and metabolites. They can be found in various types of biological fluids, including serum, saliva, urine, and nasal secretion [119]. EVs have several advantages as a potential non-invasive diagnostic and therapeutic tool. In addition to the natural enrichment of target molecules inside EVs, these molecules are protected by a lipid bilayer and are more stable than free molecules in biological fluids. These features allow ease of sample handling and result reproducibility, and thus potentially facilitate translation from bench to bedside use. Multiple studies have identified an association between the content of circulating EVs and AF [120,121]. A study by Shaihov-Teper et al. analyzed EVs derived from the epicardial fat of individuals with (n = 32) and without AF (n = 30) [122]. EVs from patients with AF contained more proinflammatory and profibrotic cytokines and profibrotic microRNAs compared to those derived from the controls. More importantly, induced pluripotent stem-derived cardiomyocytes treated with epicardial fat-derived EVs from patients with AF had a short action potential duration and had more inducible sustained re-entry, both of which are markers of AF susceptibility. Additionally, as EVs have been shown to facilitate intra- and inter-organ communication [123,124], they could participate in communication between adipose tissue and atrial tissue and thus facilitate obesity-related AF. Moreover, there is an elevated serum level of adipocyte-derived EVs in obese individuals [125]. The cargo of these EVs has been shown to facilitate diseases associated with obesity. For example, hepatocytes treated with EVs isolated from VAT of obese individuals developed dysregulation of the transforming growth factor pathway, which is known to be associated with fatty liver disease [126]. Additionally, EVs derived from adipose tissue macrophages in obese mice contain microRNAs associated with signaling pathways involving insulin resistance [127].

In addition to their diagnostic potential, EVs may prove to be an invaluable therapeutic tool. EV-mimic nanovesicles are synthetic lipid bilayers containing small molecules, drugs, or nucleic acids. The properties of these nanovesicles, such as sizes, surface charges, and surface peptides, can be adjusted to target specific organs or tissues [128]. Therapeutic agents, such as microRNAs, can then be loaded into these synthetic nanovesicles. Therefore, these unique properties make EV-mimic nanovesicles an appealing tool for targeted AF treatment.

Lastly, it is important to consider AF as a heterogenous disease, a common phenotype resulting from multiple pathologies beyond valvular versus non-valvular AF. This may also explain why current standard-of-care treatments for all types of AF fail to sustain sinus rhythm. An analogy can be drawn between AF and heart failure with preserved ejection fraction (HFpEF), which accounts for 50% of overall heart failure cases. Despite great success in clinical trials for the treatment of heart failure with reduced ejection fraction (HFrEF), the results of clinical trials for the treatment of HFpEF fail to reach a similar degree of positive outcomes. As HFpEF is composed of diverse phenotypes, future effort are directed toward phenotype-specific treatment design [129]. This framework should be adapted to future AF research such that diagnostic tools and treatments are designed to target the unique pathophysiology underlying the sub-phenotypes of AF in each patient.

## 8. Conclusions

The global burden of AF is rising with the aging of the population and its associated comorbidities. Obesity-related AF significantly contributes to this burden, as the worldwide prevalence and incidence of obesity in adults and children are also increasing at an alarming rate. Therefore, it is critical to understand the mechanisms underlying obesity-related AF. Not only will this knowledge allow discoveries of novel diagnostic/prognostic tools and more effective treatments, but it will also lead to the development of new strategies for primary and secondary AF prevention. Lastly, it is critical to emphasize the importance of primordial prevention, which includes multidisciplinary effort [130] to decrease the worldwide obesity burden.

## Data Availability

Not applicable.
